# Response to Middleton et al.

**DOI:** 10.1093/esj/aakag058

**Published:** 2026-09-07

**Authors:** Hanne Christensen, Melinda B Roaldsen, Aleš Tomek, Bjorn Logi Thórarinsson, Arlene Wilkie, Francesca R Pezzella

**Affiliations:** Department of Neurology, Copenhagen University Hospital, Copenhagen, Denmark; Clinical Research Department, University Hospital of North Norway, Tromsø, Norway; Department of Clinical Medicine, The Arctic University of Norway, Tromsø, Norway; Neurology Department, Second Medical Faculty of Charles University and University Hospital Motol and Homolka, Prague, Czech Republic; Department of Neurology, Landspitali, Reykjavík, Iceland; Stroke Alliance for Europe, London, United Kingdom; Department of Neuroscience, San Camillo-Forlanini Hospital, Rome, Italy

**Keywords:** fever, hyperglycaemia, stroke unit

Middleton et al. raise concerns that the statement “Glycaemic control in non-diabetic patients and temperature control should not be delivered to improve outcomes after stroke” from the updated version of Stroke Action Plan Europe^1^ is not supported by contemporary evidence, is based on outdated sources, and risks undermining high-value, low-cost stroke-unit care.

As to glycaemic control in acute stroke, the European Stroke Organisation (ESO) guideline recommends against the routine use of IV insulin in ischaemic stroke and intracerebral hemorrhage (ICH) to improve outcome.^2^ No other recommendations were made based on a lack of evidence. After the publication of this guideline, one RCT^3^ comparing intensive vs standard treatment of hyperglycaemia to improve outcome after stroke has been published; the findings did not support the use of intensive glycaemic control, as no control group was included in the trial, and the benefit of standard glucose control was not evaluated.

As to fever control, the ESO guideline^4^ could not make any recommendations for treatment to improve outcome due to low-quality evidence, they observed moderate evidence to suggest against routine prevention of hyperthermia with antipyretics as a means to improve functional outcome and/or survival in patients with acute ischemic stroke and normothermia. After the publication of this guideline, no RCTs have compared fever control to a placebo intervention.

The recent ESO ICH guideline,^5^ recommended against intensively controlling blood glucose as well as hyperthermia as single measures to improve outcome. The guideline further recommended implementing a care bundle, in the expert consensus statement listing the items from INTERACT3 plus three additional items, including avoiding do-not resuscitate orders within the first 24 h, dysphagia screening and treatment, and early consultation with a neurosurgeon. Furthermore, randomisation into care bundle trials was recommended.

In conclusion, our original article is supported by the best available contemporary evidence.

However, we readily agree that the strength of the evidence is low and future RCTs testing the benefits and harms of glucose and temperature control may change guidelines.

Organised stroke unit care and access to care are core issues of SAP-E. Care bundles are an attractive way of implementation, but individual items should generally show benefit in individual testing unless based on general medical knowledge, such as avoiding bed rest deconditioning. Organised stroke unit care remains a comprehensive, multidisciplinary intervention which includes numerous pharmacological and non-pharmacological interventions. We respectfully consider that this position does not compromise the foundations of high-quality stroke unit care.
